# Supplementary material for: Postoperative Imaging and Tumor Marker Surveillance in Resected Pancreatic Cancer
Source: J Clin Med. 2019 Jul 27;8(8):1115. doi: 10.3390/jcm8081115 (PMC6722558; doi:10.3390/jcm8081115)
Supplement: Supplementary file 1 [file jcm-08-01115-s001.pdf]

Table S1. Time from surgery to start of adjuvant chemotherapy in four groups.

		Symptom group (n=43)	Imaging group (n=21)	Marker group (n=42)	Intense group (n=74)	P value
**Time (weeks)§**		10.7	10.0	7.0	6.9	0.439¶
**n=**	**> 8weeks**	2	2	4	6	
**n=**	**≤ 8weeks**	1	1	11	27	
	**No adjuvant**	40	18	27	41	
**Range (weeks)**		4.0-28.6	6.0-13.6	3.4-13.6	4.0-12.3	

¶ Kruskal-Wallis test

§ Median value

Table S2. Post-operative follow-up patter in four groups.

		Symptom group (n=44)		Imaging group (n=21)		Marker group (n=42)		Intense group (n=74)	
	**Months**	**N**	**%**	**N**	**%**	**N**	**%**	**N**	**%**
**Imaging interval**	Never	2	4.5%	0	0%	4	9.5%	0	0%
	≤ 4	0	0%	21	100%	0	0%	74	100%
	4 to ≤6	12	27.3%	0	0%	17	40.5%	0	0%
	6 to ≤12	18	40.9%	0	0%	17	40.5%	0	0%
	> 12	12	27.3%	0	0%	4	9.5%	0	0%
**CA19-9 interval**	Never	11	25.0%	9	42.9%	0	0%	1	1.4%
	≤ 4	0	0%	0	0%	40	95.2%	73	98.6%
	4 to ≤6	16	36.4%	9	42.9%	1	2.4%	0	0%
	6 to ≤12	14	31.8%	3	14.3%	1	2.4%	0	0%
	> 12	3	6.8%	0	0%	0	0%	0	0%
**CEA interval**	Never	11	25.0%	11	52.4%	4	9.5%	8	10.8%
	≤ 4	0	0%	0	0%	26	61.9%	59	79.7%
	4 to ≤6	14	31.8%	6	28.6%	9	21.4%	3	4.1%
	6 to ≤12	15	34.1%	4	19.0%	2	4.8%	4	5.4%
	> 12	4	9.1%	0	0%	1	2.4%	0	0%
